# Clinical characteristics and factors associated with severe COVID-19 among hospitalized pediatric patients: a retrospective cohort study in the Chaoshan region of China

**DOI:** 10.3389/fped.2025.1627428

**Published:** 2025-08-21

**Authors:** Xianyao Wang, Ruiling Ma, Wenshan Zhong, Haipeng Lin, Hachao Zhou, Zhiwei Xiao, Shaofen Lin, Yutao Guo, Xufeng Zheng, Mingxiang Lin

**Affiliations:** ^1^ Department of Pediatrics, Shantou Central Hospital, Shantou, Guangdong, China; ^2^ Shantou University Medical College, Shantou, Guangdong, China; ^3^ Department of Radiology, Shantou Central Hospital, Shantou, Guangdong, China

**Keywords:** SARS-CoV-2, COVID-19, hospitalized children, severe disease, neurological involvement, acute necrotizing encephalitis (ANE)

## Abstract

**Background:**

Since 2019, COVID-19 has substantially impacted global public health. Although pediatric cases generally manifest with mild symptoms, severe and even fatal outcomes have occurred. Despite the decreased viral transmissibility and pathogenicity observed in the post-pandemic era, identifying early clinical indicators for severe pediatric COVID-19 remains crucial.

**Method:**

A retrospective cohort study analyzed 287 hospitalized pediatric COVID-19 patients admitted from December 2022 to August 2023. Clinical and laboratory data were compared between severe/critical and mild/moderate groups using univariable and multivariable analyses.

**Results:**

Among hospitalized patients, 82.2% were under 3 years, and severe or critical illness occurred in 32.8%. Fatigue (OR = 2.505, 95% CI: 1.359–4.615, *P* = 0.003) and hoarseness (OR = 2.781, 95% CI: 1.188–6.510, *P* = 0.018) were independent predictors of severity in multivariable analysis. In univariable analysis, elevated white blood cell counts, neutrophil percentage, procalcitonin (PCT), and reduced bicarbonate (CO₂) levels were also significantly associated with severe disease. All deaths (*n* = 3) involved children aged 1–1.5 years with acute necrotizing encephalopathy (ANE), two of whom also met diagnostic criteria for multisystem inflammatory syndrome in children (MIS-C).

**Conclusion:**

Pediatric COVID-19 hospitalizations predominantly involved children under 3 years of age. Fatigue was independently associated with severe or critical illness, potentially indicating early neurological involvement. Hoarseness was frequently observed in severe cases. Based on our cohort, particular attention may be warranted for children aged 1–1.5 years presenting with neurological symptoms, as all fatal cases (*n* = 3) in this age group were associated with acute necrotizing encephalopathy (ANE), and two were additionally complicated by multisystem inflammatory syndrome in children (MIS-C).

## 1 Introduction

Since December 2019, coronavirus disease 2019 (COVID-19) has emerged as a major global health threat (1, 2). While the pandemic has largely subsided, its residual impact persists. Fluctuating case numbers across regions underscore the ongoing threat posed by coronaviruses as potential drivers of future public health challenges (3, 4). Current evidence suggests that children are generally less susceptible to severe SARS-CoV-2 infection compared to adults (5–8); however, serious complications such as acute necrotizing encephalopathy (ANE) and multisystem inflammatory syndrome in children (MIS-C), and even fatal cases, were not uncommon during the early stage of the pandemic (9–13). Although numerous studies have described the presenting symptoms in pediatric cases and compared clinical features across severity levels (12, 14–20), the association between specific symptoms and disease severity remains inadequately defined. This study aimed to further characterize the clinical spectrum of pediatric COVID-19 and to identify risk factors associated with severe or critical illness, with the goal of providing early warning indicators to assist frontline clinicians in recognizing high-risk patients.

## 2 Materials and methods

### 2.1 Study population

**Figure 1:**
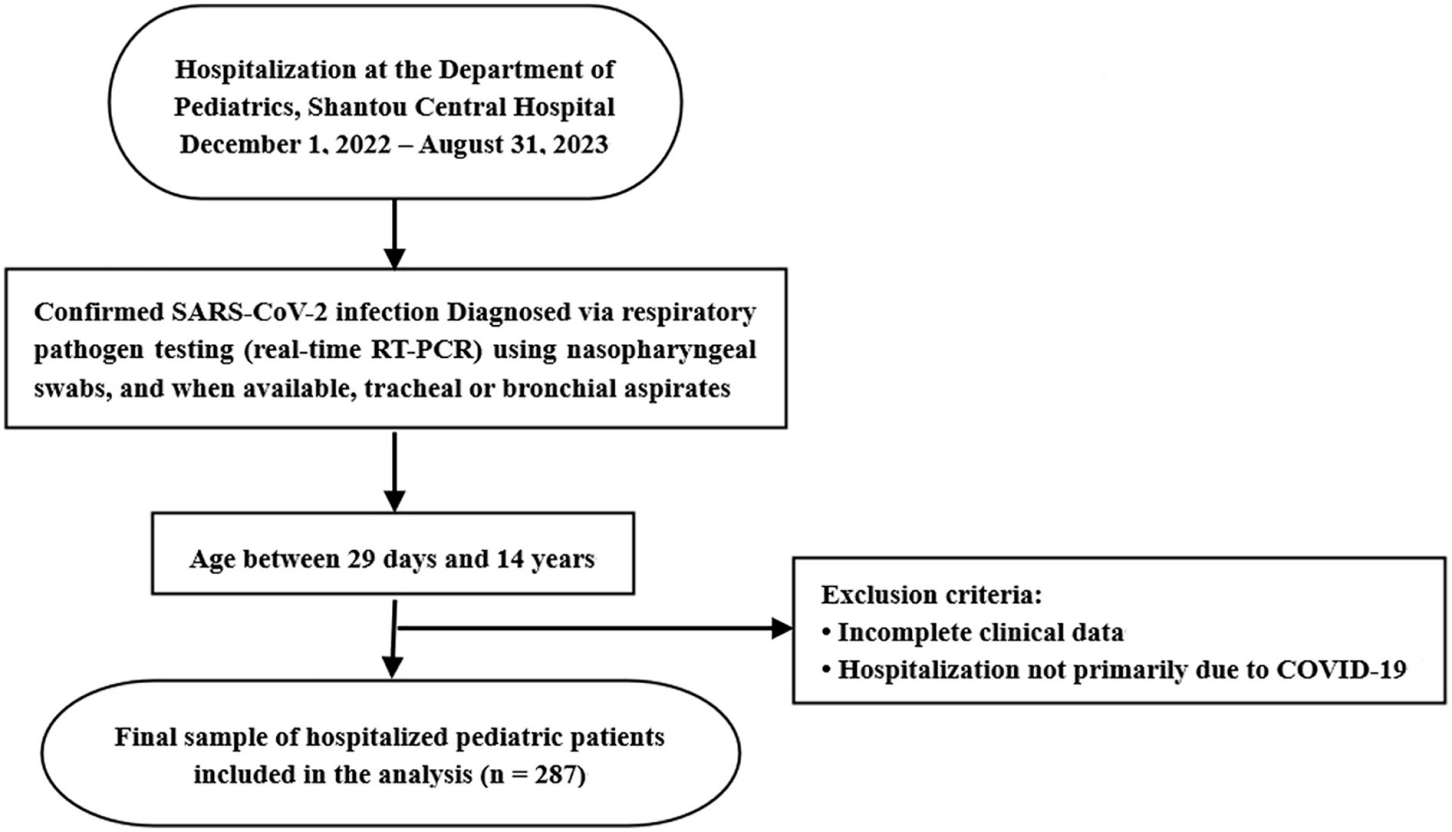
Patient selection flowchart for analysis of pediatric COVID-19 hospitalizations. This chart outlines the inclusion and exclusion process for hospitalized children with confirmed SARS-CoV-2 infection at Shantou Central Hospital between December 1, 2022 and August 31, 2023.

This retrospective, single-center study included data from 287 pediatric patients (191 boys and 96 girls), aged 2 months to 14 years (median: 11 months), with laboratory-confirmed SARS-CoV-2 infection. All patients were admitted to the Department of Pediatrics at Shantou Central Hospital, Shantou, China, between December 1, 2022, and August 31, 2023. Patients were identified through hospital records. Inclusion criteria were: (1) age between 29 days and 14 years, and (2) hospitalization primarily due to COVID-19. Patients were excluded if clinical data were incomplete or if COVID-19 was not the primary reason for admission. A total of 287 patients met the criteria and were included in the final analysis (Figure 1). The study was conducted in accordance with the Declaration of Helsinki and was approved by the Ethics Committee of Shantou Central Hospital [Ethics approval number: Scientific Research (2023) No. 070]. The requirement for informed consent was waived due to the retrospective nature of the study and the use of anonymized clinical data.

### 2.2 Laboratory methods

Respiratory specimens for SARS-CoV-2 RT-PCR testing were collected via nasopharyngeal swabs, and when available, tracheal or bronchial aspirates. Serum samples for serological testing were analyzed in the hospital's clinical microbiology laboratory using commercially available diagnostic kits. Additional hematological, biochemical, and microbiological tests were all performed within the hospital's certified laboratories using standardized and validated protocols.

### 2.3 Clinical classification of COVID-19

The classification of disease severity in this study was primarily based on the Diagnosis and Treatment Protocol for Novel Coronavirus Infection (Trial Version 10) issued by the National Health Commission (NHC) of the People's Republic of China (http://www.nhc.gov.cn/xcs/zhengcwj/202301/32de5b2ff9bf4eaa88e75bdf7223a65a.shtml) and the WHO 2023 guidelines (Clinical Management of COVID-19: Living Guideline, https://www.who.int/publications/i/item/WHO-2019-nCoV-clinical-2023.2) (21, 22). According to these guidelines, pediatric COVID-19 cases are categorized as mild, moderate, severe, or critical based on clinical symptoms, respiratory parameters, oxygen saturation, and the presence of organ dysfunction. Notably, seizures are explicitly listed as one of the clinical manifestations used to define severe disease in children. However, clinical observations in our cohort revealed that a subset of pediatric patients who experienced seizures had only simple febrile convulsions, without subsequent neurological involvement or abnormalities, and showed favorable outcomes. Based on this, and to assess the predictive value of seizures in the stratification of disease severity, we did not consider seizure occurrence alone as a direct criterion for classifying patients into the severe group. Instead, seizures were treated as an independent clinical variable in the analysis. Only patients who experienced seizures in combination with clinical or radiological evidence of encephalopathy or encephalitis (e.g., altered mental status, persistent neurological deficits, cerebrospinal fluid abnormalities, or abnormal neuroimaging findings) were classified into the severe/critical group. Patients with isolated seizures and good clinical recovery were assigned to the mild or moderate group. All other classification criteria remained consistent with the referenced guidelines.

The specific definitions used for severity grouping in this study were as follows:

Asymptomatic infection: No clinical symptoms or signs; chest imaging is normal; SARS-CoV-2 nucleic acid is detected from respiratory or other specimens.

Mild type: Symptoms of upper respiratory tract infection (e.g., fever, fatigue, cough, sore throat, runny nose, sneezing) without abnormal lung findings. Some cases may present only with gastrointestinal symptoms. There are no signs of lower respiratory tract involvement on physical examination.

Moderate type: Evidence of pneumonia with frequent fever and cough. Some patients may have wheezing but no signs of significant hypoxemia (SpO₂ > 93%) or shortness of breath (RR < 30/min). In some cases, clinical signs may be absent, but imaging reveals subclinical pulmonary lesions.

Severe type: Meets any of the following criteria:

1. Hyperpyrexia or persistent high-grade fever lasting more than 3 days;
2. Shortness of breath — RR ≥ 60/min (<2 months), ≥50/min (2–12 months), ≥40/min (1–5 years), ≥30/min (>5 years), excluding fever and crying.
3. SpO₂ ≤ 93% at rest.
4. Signs of respiratory distress (e.g., moaning, nasal flaring, chest retractions, cyanosis, intermittent respiratory pauses).
5. Impaired consciousness or seizures with encephalopathy or encephalitis.
6. Feeding difficulties or refusal to eat accompanied by signs of dehydration.
7. Imaging showing bilateral or multi-lobar infiltrates, rapid lesion progression >50% in a short period, or pleural effusion.

Critical type: Meets any of the following criteria:

1. Respiratory failure requiring mechanical ventilation.
2. Shock.
3. Acute encephalopathy, acute necrotizing encephalopathy (ANE) or severe organ dysfunction requiring intensive care.

### 2.4 Statistical analyses

Statistical analyses were performed using SPSS version 26.0 (IBM Corp., Armonk, NY, USA). Continuous variables following a normal or approximately normal distribution were expressed as mean ± standard deviation (SD) and compared using independent sample t-tests. For non-normally distributed variables, data were presented as median and interquartile range (IQR), and the Mann–Whitney U test was used for between-group comparisons. Categorical variables were described as frequencies (percentages) and analyzed using chi-square tests, with continuity correction or Fisher's exact test applied when appropriate. A multivariate binary logistic regression model was constructed to identify independent predictors of severe or critical disease. All candidate variables were simultaneously entered into the model using forced-entry (Enter) method. Adjusted odds ratios (aORs) and 95% confidence intervals (CIs) were calculated to evaluate the strength of associations. A two-tailed *p*-value < 0.05 was considered statistically significant. Seizure was analyzed as an independent clinical variable and was not used to define disease severity unless accompanied by clinical or radiological evidence of encephalopathy or encephalitis, as specified in the severity grouping criteria.

## 3 Results

### 3.1 Study population and baseline characteristics

**Figure 2:**
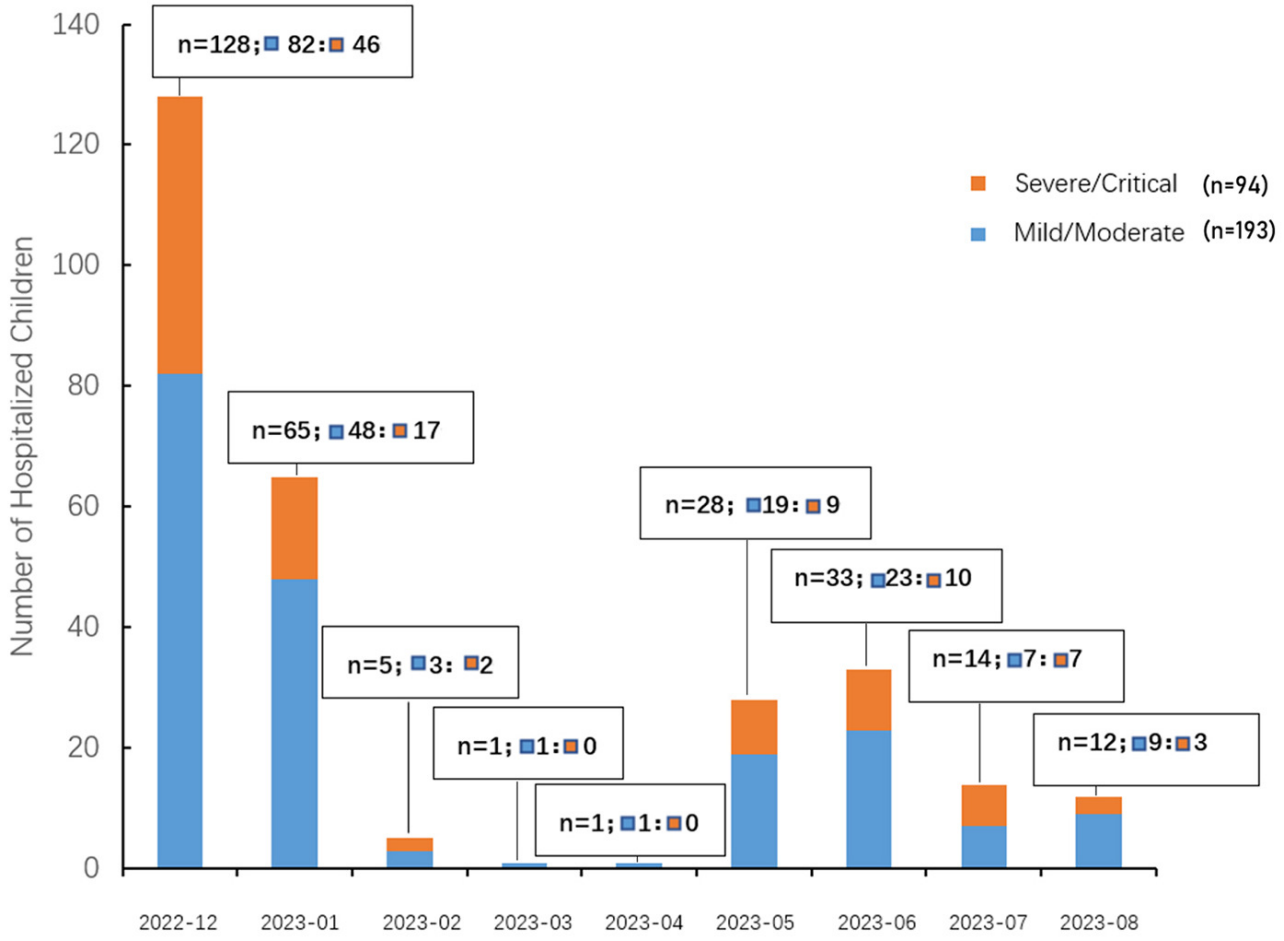
Monthly distribution of hospitalized pediatric COVID-19 cases by disease severity. The bar chart illustrates the number of pediatric patients hospitalized with COVID-19 from December 2022 to August 2023, stratified by disease severity. Orange bars represent children classified as severe/critical, while blue bars represent those with mild/moderate disease. Each bar is labeled with the total number of hospitalized children (*n*), and the breakdown of mild/moderate (▪) and severe/critical (▪) cases.

**Figure 3:**
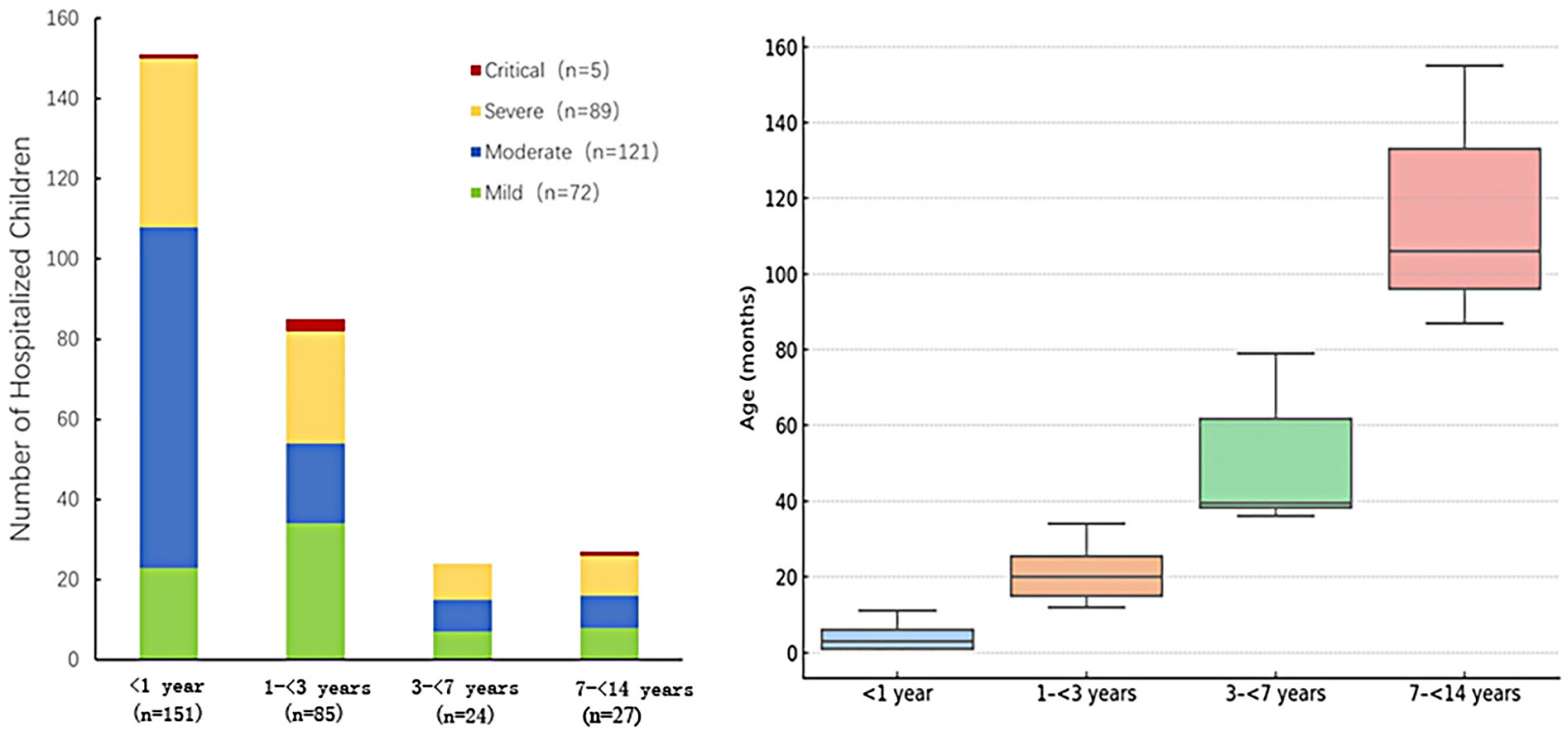
Distribution of disease severity among hospitalized pediatric patients stratified by age group (*N* = 287). Left: The stacked bar chart displays the number of hospitalized children categorized by disease severity (Mild, Moderate, Severe, and Critical) across four age groups: <1 year (*n* = 151), 1 to <3 years (*n* = 85), 3 to <7 years (*n* = 24), and 7 to <14 years (*n* = 27). The severity levels are represented by different colors: Mild (green, *n* = 72), Moderate (blue, *n* = 121), Severe (yellow, *n* = 89), and Critical (red, *n* = 5). Right: box plot representing the distribution of age (in months) within each age group. The median and interquartile range are displayed.

A total of 287 pediatric patients with COVID-19 were enrolled in this study, including 191 males (66.6%) and 96 females (33.4%), yielding a male-to-female ratio of approximately 2:1 (*P* < 0.001, chi-square test against a 1:1 ratio). The median age was 11.2 months [interquartile range (IQR): 3.23–26 months] (Table 1). The monthly distribution of hospitalized pediatric cases is shown in Figure 2. A peak in hospital admissions occurred in December 2022, with 128 cases reported: 82 classified as mild/moderate and 46 as severe/critical. This was followed by a gradual decline in January 2023 (*n* = 65), and a sharp drop in February (*n* = 5) and March (*n* = 1). A secondary rise in cases was observed in May (*n* = 28), June (*n* = 33), and July (*n* = 14), with both mild and severe cases represented (Figure 2). Hospitalized pediatric patients were predominantly under 3 years of age (82.2%), particularly those younger than 1 year, accounting for 52.6% (151/287) of total admissions; patients younger than 3 years accounted for 82.2% (236/287) of all admissions (Figure 3). The overall median length of hospital stay was 5 days (IQR: 3–6 days), with a mean duration of 5.2 days. A total of 35 children (12.2%) required admission to the pediatric intensive care unit (PICU). Among these PICU patients, the median hospital stay was longer, at 7 days (IQR: 4.5–9.5 days), with a mean of 7.6 days.

**Table 1:** Comparison of demographic, clinical, and laboratory characteristics between pediatric patients with mild/moderate and severe/critical disease.

Category	Parameter	Overall (*N* = 287)		Classification		*χ*^2^ or *Z*/*t*	*P*-value
				Mild/Moderate (*n* = 193) (67.2%)*	Severe/Critical x(*n* = 94) (32.8%)*		
Demographics	Age	11.2 [3.23, 26]		9.43 [2.40, 25.00]	13.00 [6.47, 28.00]	−2.304 (*Z*)	0.021
	Gender	Male	191 (66.6%)	129 (66.8%)	62 (66.0%)	0.022 (*χ*^2^)	0.882
		Female	96 (33.4%)	64 (33.2%)	32 (34.0%)		
Clinical Signs	Seizures	No	200 (69.7%)	139 (72.0%)	61 (64.9%)	1.520 (*χ*^2^)	0.218
		Yes	87 (30.3%)	54 (28.0%)	33 (35.1%)		
	Fatigue/Weakness	No	204 (71.1%)	150 (77.7%)	54 (57.4%)	12.639 (*χ*^2^)	0.001
		Yes	83 (28.9%)	43 (22.3%)	40 (42.6%)		
	Hoarseness/Voice Changes	No	254 (88.5%)	178 (92.2%)	76 (80.9%)	8.040 (*χ*^2^)	0.005
		Yes	33 (11.5%)	15 (7.8%)	18 (19.1%)		
	Diarrhea	No	273 (95.1%)	185 (95.9%)	88 (93.6%)	0.285 (*χ*^2^)	0.593
		Yes	14 (4.9%)	8 (4.1%)	6 (6.4%)		
	Vomiting	No	230 (80.1%)	160 (82.9%)	70 (74.5%)	2.825 (*χ*^2^)	0.093
		Yes	57 (19.9%)	33 (17.1%)	24 (25.5%)		
	Conjunctivitis	No	282 (98.3%)	191 (99%)	91 (96.8%)	0.687 (*χ*^2^)	0.407
		Yes	14 (4.9%)	2 (1%)	3 (3.2%)		
	Rash	No	273 (95.1%)	189 (97.9%)	88 (93.6%)	2.328 (*χ*^2^)	0.127
		Yes	5 (1.7%)	4 (2.1%)	6 (6.4%)		
	Underlying Diseases	No	247 (86.1%)	169 (87.6%)	78 (83%)	1.108 (*χ*^2^)	0.292
		Yes	40 (13.9%)	24 (12.4%)	16 (17%)		
Laboratory	WBC (×10⁹/L)	7.8 (5.68, 10.76)		7.60 (5.83,10.05)	9.12 (5.92,12.07)	−2.237(*Z*)	0.025
	Neutrophils (×10⁹/L)	3.6 (2.1, 5.5)		3.60 (2.10,4.90)	4.40 (2.60,7.30)	−3.091(*Z*)	0.002
	Lymphocytes (×10⁹/L)	2.65 (1.4, 4.5)		2.80 (1.40,4.90)	2.20 (1.30,3.80)	−1.003(*Z*)	0.316
	Platelets (×10⁹/L)	268 (213.75, 349)		273.00 (213.00,350.00)	261.00 (208.00,340.00)	−0.405(*Z*)	0.686
	CRP (mg/L)	1.78 (0.04, 12.7)		1.24 (0.04,12.10)	6.08 (0.04,16.37)	−1.238(*Z*)	0.216
	PCT (ng/ml)	0.2 (0.13, 0.48)		0.18 (0.12,0.35)	0.30 (0.17,0.88)	−3.912(*Z*)	0.001
	ALT (U/L)	23 (17, 32)		23.00 (17.00,31.00)	23.00 (17.00,33.00)	−0.383(*Z*)	0.701
	AST (U/L)	48.5 (39, 61.25)		48.00 (39.00,61.00)	50.00 (37.00,62.00)	−0.157(*Z*)	0.876
	CKMB (U/L)	28 (21, 37.5)		28.00 (22.00,36.00)	27.00 (20.00,41.00)	−0.082(*Z*)	0.935
	LDH (U/L)	322 (285, 379)		324.00 (291.00,378.00)	310.00 (278.00,390.00)	−0.941(*Z*)	0.347
	CO2 (mmol/L)	20.4 (18.4, 22.03)		20.50 (18.70,22.40)	20.10 (18.10,21.50)	−1.976(*Z*)	0.048
	Hb (g/L)	116.65 ± 12.96		116.47 ± 13.05	117.02 ± 12.83	−0.335 (*t*)	0.738

Data are presented as number (%), median (IQR), or mean ± standard deviation. Percentages marked with an asterisk (*) indicate proportions calculated based on the total study population (*N* = 287); all other percentages are based on the number of patients within each severity group (mild/moderate, *n* = 193; Statistical comparisons were performed using Chi-square test (*χ*^2^) for categorical variables, Mann–Whitney U test (*Z*) for continuous variables with non-parametric distribution [median (IQR)], and Student's *t*-test (*t*) for continuous variables with parametric distribution (mean ± SD). Seizure was analyzed as an independent variable and was not used to define disease severity unless accompanied by encephalopathy or encephalitis.

### 3.2 Clinical presentations and laboratory findings

Among the 287 patients, seizures occurred in 87 (30.3%), fatigue or weakness in 83 (28.9%), and hoarseness or voice changes in 33 (11.5%). Other reported symptoms included vomiting in 57 (19.9%), underlying chronic diseases in 40 (13.9%), diarrhea in 14 (4.9%), rash in 10 (3.5%), and conjunctivitis in 5 (1.7%). Laboratory findings are summarized in Table 1, with complete data presented therein.

### 3.3 Clinical and laboratory characteristics stratified by disease severity

**Figure 4:**
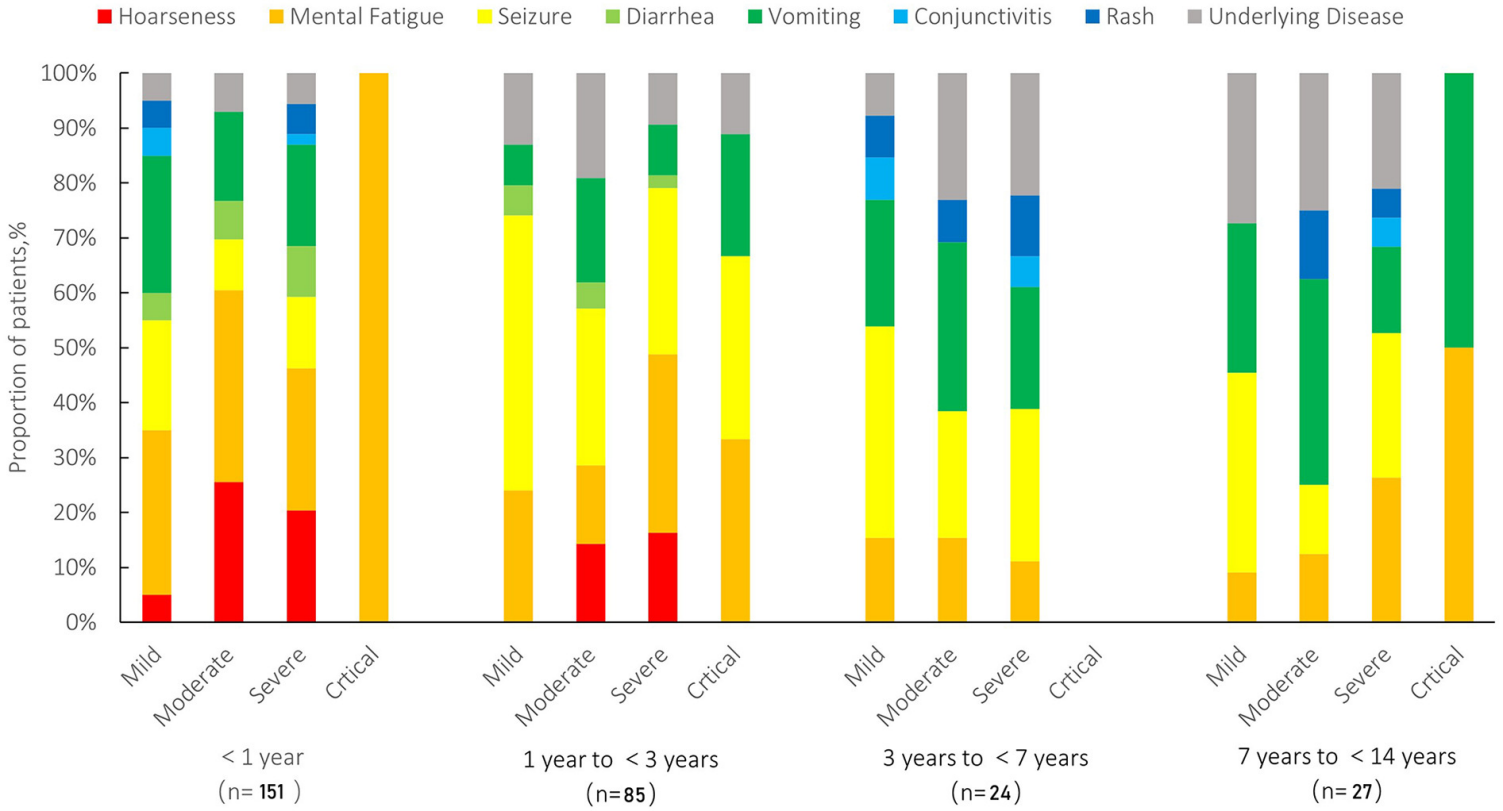
Distribution of clinical symptoms stratified by age and disease severity in pediatric patients (*N* = 287). Symptoms include hoarseness (red), mental fatigue (orange), seizure (yellow), diarrhea (light green), vomiting (green), conjunctivitis (light blue), rash (blue), and underlying diseases (gray). Patients were categorized into four age groups (<1 year, 1 to <3 years, 3 to <7 years, and 7 to <14 years) and further stratified by disease severity (mild, moderate, severe, and critical).

Among the 287 hospitalized pediatric patients, 25% (*n* = 72) were classified as mild, 42% (*n* = 121) as moderate, 31% (*n* = 89) as severe, and 2% (*n* = 5) as critical. The age distribution across different severity groups is further illustrated in Figure 3. Figure 3A (left) demonstrates that among infants, moderate cases were the most frequent, while the 1–<3-year age group had the highest number of critical cases (*n* = 3). Figure 3B (right) presents box plots of age (in months) stratified by severity group, showing that age distributions were generally symmetrical within each group and that no significant outliers were observed. Together, these visualizations support the demographic concentration of pediatric COVID-19 hospitalizations in early childhood. The median age of patients in the mild/moderate group was significantly lower [9.43 months (IQR: 2.40–25.00)] compared to the severe/critical group [13.00 months (IQR: 6.47–28.00), *Z* = −2.304, *P* = 0.021]. Gender distribution showed no statistically significant difference between the groups (*χ*^2^ = 0.022, *P* = 0.882). Regarding clinical manifestations, fatigue or weakness was significantly more common in the severe/critical group (42.6%) compared to the mild/moderate group (22.3%) (*χ*^2^ = 12.639, *P* = 0.001). Similarly, hoarseness or voice changes were significantly more prevalent in the severe/critical group (19.1%) than in the mild/moderate group (7.8%) (*χ*^2^ = 8.040, *P* = 0.005). No significant differences were found between the groups for seizures, diarrhea, vomiting, conjunctivitis, rash, or underlying diseases (all *P* > 0.05) (Table 1). Among different age groups (<1 year, 1 to <3 years, 3 to <7 years, and 7 to <14 years), symptoms such as hoarseness, fatigue, and seizures were more prominent in younger children (<1 year), particularly in severe and critical cases. In contrast, diarrhea, vomiting, and rash were more prevalent in older children (≥3 years). Underlying chronic diseases (*N* = 40) were observed across all age groups, with a higher proportion noted among older children (Figure 4). Laboratory results indicated significantly higher white blood cell counts [9.12 (IQR: 5.92–12.07) × 10⁹/L vs. 7.60 (IQR: 5.83–10.05) × 10⁹/L, *Z* = −2.237, *P* = 0.025], neutrophil counts [4.40 (IQR: 2.60–7.30) × 10⁹/L vs. 3.60 (IQR: 2.10–4.90) × 10⁹/L, *Z* = −3.091, *P* = 0.002], procalcitonin [0.30 (IQR: 0.17–0.88) ng/ml vs. 0.18 (IQR: 0.12–0.35) ng/ml, *Z* = −3.912, *P* = 0.001], and CO₂ levels [20.10 (IQR: 18.10–21.50) mmol/L vs. 20.50 (IQR: 18.70–22.40) mmol/L, *Z* = −1.976, *P* = 0.048] in the severe/critical group compared with the mild/moderate group. No statistically significant differences were observed in lymphocyte counts, platelets, CRP, ALT, AST, CKMB, LDH, or hemoglobin (Hb) levels between groups (all *P* > 0.05) (Table 1).

### 3.4 Multivariate logistic regression analysis of independent predictors associated with severe or critical disease

Multivariate logistic regression analysis was conducted to identify independent factors associated with severe or critical disease (Table 2). After adjustment for confounding variables, fatigue or weakness (OR: 2.505, 95% CI: 1.359–4.615, *P* = 0.003) and hoarseness (OR: 2.781, 95% CI: 1.188–6.510, *P* = 0.018) were identified as significant predictors for severe or critical disease among pediatric patients. However, age, white blood cell (WBC) count, neutrophil count, procalcitonin (PCT), and carbon dioxide (CO₂) levels were not significantly associated with disease severity (all *P* > 0.05).

**Table 2:** Multivariate logistic regression analysis of factors associated with severe or critical disease.

Variable	*β*	SE	Wald	*P*	OR	95% CI for OR	
						Lower limit	Upper limit
Age (months)	0.005	0.005	0.929	0.335	1.005	0.995	1.015
Fatigue/Weakness	0.918	0.312	8.672	0.003	2.505	1.359	4.615
Hoarseness/	1.023	0.434	5.559	0.018	2.781	1.188	6.510
WBC	−0.048	0.061	0.622	0.430	0.953	0.847	1.074
Neutrophil count	0.152	0.086	3.092	0.079	1.164	0.983	1.378
PCT	0.165	0.149	1.222	0.269	1.179	0.881	1.579
CO2	−0.055	0.053	1.087	0.297	0.947	0.854	1.049
Constant	−0.418	1.145	0.133	0.715	0.658		

*β*, regression coefficient; SE, standard error; OR, odds ratio; CI, confidence interval; WBC, white blood cell count; PCT, procalcitonin; CO₂, carbon dioxide. Variables with *P* < 0.05 are considered statistically significant.

### 3.5 Oxygen therapy

A total of 47 pediatric patients (16.4% of 287 patients) required oxygen therapy. Among these, 33 (70.2%) received low-flow oxygen therapy, 9 (19.2%) received mask oxygen therapy, and 5 (10.6%) required mechanical ventilation (Table 3). Comparison of demographic characteristics showed no significant difference in median age or gender distribution between oxygen and non-oxygen therapy groups (*P* = 0.347 and *P* = 0.268, respectively). Clinical symptom analysis indicated that seizures and fatigue/weakness were significantly more prevalent among patients receiving oxygen therapy compared to those not requiring oxygen therapy (44.7% vs. 27.5%, *P* = 0.019 and 55.3% vs. 23.8%, *P* = 0.001, respectively). However, other clinical symptoms, including hoarseness, diarrhea, vomiting, conjunctivitis, rash, and presence of chronic or underlying diseases, showed no statistically significant differences between the two groups (all *P* > 0.05) (Table 3).

**Table 3:** Comparison of demographics and clinical symptoms according to oxygen therapy.

Variable	Category	No oxygen therapy (*n* = 240)	Oxygen therapy (*n* = 47)	*χ* ^2^	*P*-value
Oxygen therapy					
Oxygen therapy*		240 (83.6%)	47 (16.4%)		
Low-flow oxygen		–	33 (70.2%)		
Mask oxygen		–	9 (19.2%)		
MV		–	5 (10.6%)		
Demographics					
Median age (months)		10.73 (3.57,28.00)	11.27 (2.10,21.00)	−0.941	0.347
Male		163 (67.9%)	28 (59.6%)	1.229	0.268
Female		77 (32.1%)	19 (40.4%)		
Clinical symptoms					
Seizures	No	174 (72.5%)	26 (55.3%)	5.492	0.019
	Yes	66 (27.5%)	21 (44.7%)		
Fatigue/Weakness	No	183 (76.3%)	21 (44.7%)	19.055	0.001
	Yes	57 (23.8%)	26 (55.3%)		
Hoarseness	No	213 (88.8%)	41 (87.2%)	0.089	0.766
	Yes	27 (11.3%)	6 (12.8%)		
Diarrhea	No	228 (95%)	45 (95.7%)	0.000	1.000
	Yes	12 (5%)	2 (4.3%)		
Vomiting	No	191 (79.6%)	39 (83%)	0.285	0.594
	Yes	49 (20.4%)	8 (17%)		
Conjunctivitis	No	235 (97.9%)	47 (100%)	∼	1.000
	Yes	5 (2.1%)	0 (0%)		
Rash	No	231 (96.3%)	46 (97.9%)	0.014	0.905
	Yes	9 (3.8%)	1 (2.1%)		
Underlying diseases	No	208 (86.7%)	39 (83%)	0.446	0.504
	Yes	32 (13.3%)	8 (17) %		

Data are presented as numbers, percentages (%), and medians with interquartile ranges (IQR). Values marked with an asterisk * is calculated based on the total study population (*N* = 287); all other percentages are calculated based on the number of patients in each group (no oxygen therapy, *n* = 240; oxygen therapy, *n* = 47). Statistical comparisons were performed using Chi-square test (*χ*^2^) for categorical variables and Mann–Whitney U test (*Z*) for continuous variables. MV, mechanical ventilation.

### 3.6 Clinical characteristics and prognosis of 5 pediatric patients with critical-type COVID-19

**Figure 5:**
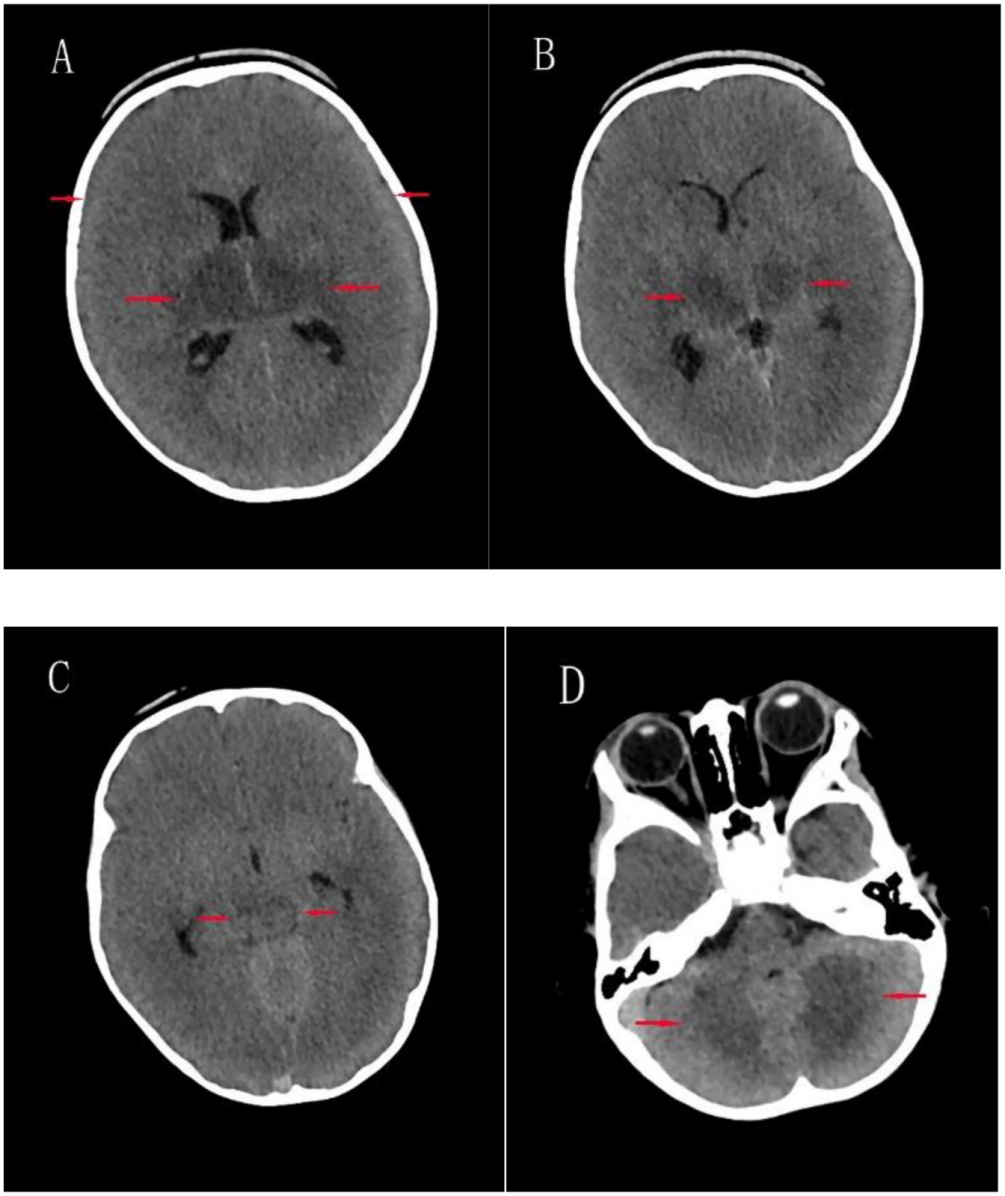
Axial brain CT images of case 2. **(A)** Bilateral cerebral hemispheric swelling with effacement of sulci and fissures, blurred gray-white matter differentiation, and mildly hypodense areas in the bilateral basal ganglia; **(B)** mild hypodensity in the bilateral thalami; **(C)** Mild hypodensity in the upper midbrain; **(D)** Patchy mild hypodensities in the bilateral cerebellar hemispheres.

Detailed clinical features, laboratory findings, and outcomes of five pediatric patients (Case 1–5) diagnosed with critical-type COVID-19 are summarized in Table 4. These critically ill patients ranged in age from 37 days to over 11 years and included three males and two females. All five patients presented with fatigue and weakness. Seizures were reported in three of the five cases (60%), and vomiting occurred in three patients as well, with two experiencing both symptoms. None of the patients presented with diarrhea, rash, or conjunctivitis. Laboratory investigations revealed significantly elevated levels of D-dimer (360–50, 740 ng/ml), lactate (2.06–8.91 mmol/L), and highly variable procalcitonin concentrations (0.084–50 ng/ml). Notably, marked elevations in D-dimer (50,740 and 16,900 ng/ml) and interleukin-6 (>4,000 and 2,141 pg/ml) were observed in two patients. Troponin-I levels were increased in two patients (maximum 0.468 ng/ml). Carbon dioxide (CO₂) levels ranged from critically low (11.9 mmol/L) to normal (24 mmol/L). Multisystem inflammatory syndrome in children (MIS-C) was diagnosed in two of the five critical cases (40%), and three patients (60%) developed acute necrotizing encephalopathy (ANE). All five critical cases required mechanical ventilation via endotracheal intubation. The three ANE cases, all of whom died, occurred in children aged between 1 and 1.5 years and accounted for a mortality rate of 1.05% in the overall cohort. An axial brain CT image from Case 2 is shown in Figure 5, demonstrating extensive brain injury consistent with clinical features suggestive of ANE.

**Table 4:** Clinical features, laboratory findings, and outcomes of five pediatric patients with critical COVID-19.

Variable	Case 1	Case 2	Case 3	Case 4	Case 5
Age	18 months	17 months	13 months	11+ years	37 days
Gender	Male	Male	Female	Female	Male
Seizure	+	+	+	–	–
Fatigue/Weakness	+	+	+	+	+
Diarrhea	–	–	–	–	–
Hoarseness	–	–	–	–	–
Vomiting	–	+	+	+	–
Conjunctivitis	–	–	–	–	–
Rash	–	–	–	–	–
Underlying Diseases	–	–	IEI *	–	–
D-D	50,740	16,900	5,050	360	960
LAC	4.98	8.91	5.63	2.06	5.22
PCT	12.5	50	0.084	0.257	0.35
CRP	<5	<5	<5	16.3	<5
IL-6	>4,000	2,141	<4	145	<4
CO2	12.3	11.9	17.2	19.9	24
TNI	0.468	0.307	0.004	0.005	/
MIS-C (Y or N)	Y	Y	N	N	N
ANE (Y or N)	Y	Y	Y	N	N
MV (ETI)	Y	Y	Y	Y	Y
Outcomes	Death	Death	Death	Recovered	Recovered

“+” indicates the presence of a symptom; “–” indicates its absence; “/” indicates missing data. IEI, inborn errors of immunity. The patient presented with scattered hyperpigmented macules on the skin shortly after birth. Genetic testing identified a heterozygous variant in the *IKBKG* gene: NM_001099857.1:c.1110delinsTT (p.Ala371Cysfs24). The clinical phenotype was suggestive of incontinentia pigmenti, immunodeficiency 33, and ectodermal dysplasia with immunodeficiency 1. D-D, D-dimer (ng/ml); LAC, lactate (mmol/L); PCT, procalcitonin (ng/ml); CRP, C-reactive protein (mg/L); IL-6, interleukin-6 (pg/ml); CO₂, carbon dioxide (mmol/L); TNI, troponin I (ng/ml); MIS-C, multisystem inflammatory syndrome in children; ANE, acute necrotizing encephalopathy; ETI, endotracheal intubation.

## 4 Discussion

In this single-center cohort study, we analyzed 287 pediatric patients hospitalized with COVID-19. Most of the patients were under 3 years of age (82.2%), with infants (<1 year) accounting for 52.6%. Among infants, moderate illness was the most frequently observed. Severe and critical illness occurred in 32.8% of the total cohort. Among the five children who were critically ill, three died, representing 1.05% of the total cases. All three fatalities occurred in children aged between 1 and 1.5 years. The overall male-to-female ratio was 2:1, which showed a statistically significant difference; however, no sex-related differences were observed across categories of disease severity. In terms of age distribution, infants and toddlers—particularly infants—accounted for the highest proportion of pediatric COVID-19 hospitalizations. This observation is consistent with previous epidemiological data. For example, a study conducted in the United States reported that nearly 44% of children hospitalized with COVID-19 were infants younger than 6 months (23–25). In terms of disease severity by age, moderate illness was most frequently observed in infants, while toddlers primarily exhibited mild to moderate symptoms (5–8). These observations align with previous reports indicating that COVID-19 in infants and toddlers is generally mild and associated with favorable outcomes. Nevertheless, the concentration of fatal cases in the 1–1.5-year age range may suggest a potentially increased susceptibility to disease progression in this age group. This may suggest a need for closer clinical observation and timely support in children at this developmental stage. In terms of sex distribution, we observed that a significantly higher proportion of hospitalized pediatric were male compared to female (approximately 2:1). However, no significant association was found between sex and disease severity, suggesting that once hospitalized for COVID-19, boys and girls tend to experience comparable levels of clinical severity. This indicates that, based on our cohort, sex does not appear to influence clinical prognosis in pediatric COVID-19 cases. The underlying reasons for the observed sex disparity in hospitalization remain unclear. Similar conclusions have been reported in the literature, with comparable trends also observed in adult populations (26–28). In contrast, other studies have reported no significant sex-based differences in infection rates (25, 29–31). Some researchers have proposed that sex differences in the expression of hormone-regulated receptors—such as transmembrane serine protease 2 (TMPRSS2) and angiotensin-converting enzyme 2 (ACE2)—may contribute to differential susceptibility to viral entry and infection (32, 33). Alternatively, the observed imbalance might be influenced by regional differences in the sex distribution of the pediatric population. These underlying mechanisms warrant further investigation.

We found that certain clinical symptoms and laboratory findings were associated with increased disease severity. In univariate analyses, patients in the severe/critical group, compared to those in the mild/moderate group, showed significant differences in age; clinical symptoms such as fatigue/ weakness and hoarseness; and laboratory parameters, including elevated white blood cell (WBC) count, neutrophil count, and procalcitonin (PCT) levels, as well as decreased carbon dioxide (CO₂) levels. However, in the multivariate model, neither age nor laboratory markers retained statistical significance, whereas fatigue/weakness and hoarseness remained independently associated with severe illness. This may be due to the inherently low specificity of routine laboratory indicators for viral infections. In the early stages of disease, such markers may have limited sensitivity and specificity in differentiating disease severity. Notably, fatigue/weakness and hoarseness remained significantly associated with severe/critical illness even after adjusting for potential confounders in multivariate regression analysis. Fatigue/weakness may represent an early clinical manifestation of multisystem involvement, particularly of the central nervous system. Hoarseness, on the other hand, may reflect laryngeal edema, hypoxemia, or more severe respiratory tract involvement, which could explain its stronger association with severe disease. These findings suggest that specific clinical features may be more valuable than routine laboratory tests for the early prediction of disease severity in pediatric COVID-19. Among critically ill patients, all three fatal cases were complicated by acute necrotizing encephalopathy (ANE), and two developed multisystem inflammatory syndrome in children (MIS-C). MIS-C is a hyperinflammatory syndrome and a critical manifestation of COVID-19, often characterized by persistent fever, shock, and multiorgan dysfunction, and typically requires admission to a pediatric intensive care unit (PICU). According to existing literature, the median age at MIS-C onset is around 8–9 years; however, both MIS-C cases in our study occurred in the toddler age group, indicating that clinicians should maintain a high index of suspicion for MIS-C even in younger children presenting with compatible inflammatory features (34–36). ANE is another severe neurological complication of COVID-19, previously reported in several pediatric cases, with a mortality rate exceeding 50% (10, 37–41). In our study, all three ANE cases were fatal, further underscoring that central nervous system involvement is a key pathophysiological feature of severe pediatric COVID-19, particularly among critically ill children in the infant and toddler age group.

In the analysis of oxygen supplementation, 16.4% of children with COVID-19 required oxygen therapy. Children presenting with clinical features such as seizures and fatigue/weakness had a higher frequency of oxygen use compared to asymptomatic cases. This suggests that beyond respiratory involvement, which typically necessitates oxygen support, neurologic symptoms may also be associated with an increased need for oxygen therapy in pediatric COVID-19 cases. When seizures were analyzed separately in relation to disease severity classification, no statistically significant differences were found in either chi-square or multivariate regression analyses. This may be due to the high proportion of febrile seizures in clinical practice, which are generally benign and have favorable outcomes. These findings indicate that seizures alone may have limited specificity for predicting severe or critical illness. Nevertheless, in severe and especially critical cases, seizures often reflect direct neurological involvement. Clinicians should remain vigilant in assessing children with seizure episodes, particularly those who present with postictal impaired consciousness or signs of encephalitis or encephalopathy.

This study was retrospective in design, conducted at a single center, and included a relatively small number of critical cases, which may have limited the identification of certain risk factors. In addition, symptom documentation may have been subject to bias, and underlying conditions and long-term outcomes were not comprehensively assessed. Despite these limitations, our findings offer valuable insights into clinical predictors of disease severity in pediatric COVID-19, particularly for high-risk groups such as infants and toddlers.

In summary, children under 3 years of age accounted for the majority of pediatric COVID-19 hospitalizations, with infants particularly affected. Fatigue or generalized weakness was significantly associated with severe or critical illness and may represent an early, atypical sign of neurological involvement. Although hoarseness was more common in severe cases, it was not observed among those with critical illness, suggesting its limited role in predicting extreme disease. All fatalities occurred in children aged 1–1.5 years and were characterized by acute necrotizing encephalopathy (ANE), with two cases also complicated by multisystem inflammatory syndrome in children (MIS-C). These findings highlight the importance of recognizing subtle neurologic symptoms early in the disease course, particularly in younger children at higher risk of severe outcomes.

## Data Availability

The original contributions presented in the study are included in the article/Supplementary Material, further inquiries can be directed to the corresponding author.
